# The Filter-Passing Virus

**Published:** 1919-09-13

**Authors:** 


### THE FILTER-PASSING VIRUS.

On a later page we review the report by Dr. Hamer, County Medical Officer of Health for London, on the recent outbreaks of influenza, and possibly the chief interest to be derived from his writing is the clear indication of a close and definite relationship between cerebro-spinal fever, poliomyelitis, and influenza, so-called. It is but a short time ago that it was claimed in a series of much discussed papers that six diseases, including trench fever and influenza, have been conclusively shown to be caused by organisms which can pass filters of such density that they hold back all ordinary bacteria, and that in the case of these and several other diseases the virus itself has been cultivated and readily recognised under the microscope. Still more recently, from the pen of Dr. J. A. Arkwright, of the Lister Institute, comes a very severe criticism of these claims.

It is not our intention to enter into the arguments or explanations which are thus automatically suggested, but to indicate rather that, just as the efforts to associate the earlier influenza manifestations with "botulism," though proved to be misdirected, were incidentally of considerable assistance in linking up parts of the complicated history of influenzal epidemiology in England and elsewhere, so the work on the filter-passing virus will probably bear fruit before a long period has elapsed, even if the conclusions at first drawn are now considered erroneous. It is in the actual cultures and sub-cultures of the causal organisms that the failure has occurred, and as, according to all the older accepted maxims of bacteriological investigations, the causal organism of a disease must be isolated, cultivated, and reinoculated to produce the disease in the individual, a break in this chain appears, at first sight, to cause breakdown of the whole investigation, but actually (call the filter-passing unit what we will, virus toxin, or organism), the existence of some such substance is very definitely established, and it now remains to prove its degree of association with the already well-known bacteria, in these instances particularly with the gram negative diplococci and the influenzal type of bacilli.

As far as the extraordinary catarrhal outbreaks in England were concerned, a great number of eminent men maintained throughout that no *new* organisms were concerned in the disease, and our own impression has continuously held along the same lines. Dr. Hamer's report appears definitely to agree with such views, although he deals with the broad aspect of the subject and largely avoids technical details. As we have several times previously suggested, there appears to be every reason to treat the scourge of the past year as a chapter in a story which has been running for many, many ages. We know that in recent years the origin of epidemics has frequently been traced to most sparsely inhabited parts of the world and then, as in a pond whose surface has been broken by a stone thrown into it, waves travel outwards to the edges and are reflected back again. To follow this parallel in the latest world outbreaks is fascinating in itself, and Dr. Hamer has done much to introduce his readers to the study.

The interest we must take in the subject does not, however, by any means end with the explanations of waves and prevalence. The whole merges into the questions of so-called "colds," or more accurately infective catarrhs, the rival theories that exposure is the first cause or that an acute attack is always due to a fresh infection, the carrier theories and a host of but half-understood complications. It appears perhaps a long cry from the filter-passing virus to the common cold in the head, but it is worthy of note that the series of subjects already mentioned have an extremely close relationship among themselves, may well form a unit in research investigations, and must be attacked in future both frontally and at the most outlying points. The work already done, the much-criticised papers of Bradford, Bashford, and Wilson, if falling very far short of the ideal results, with which they were once, in certain quarters, credited, must be by no means discarded. The proportion of accuracy and discovery in this and a number of parallel war-time works must be carefully preserved and woven into a whole, which should in years ahead throw great light on some of the apparently simplest, but yet frequently fatal conditions with which almost every household is sooner or later beset.

